# Machine learning to support citizen science in urban environmental management

**DOI:** 10.1016/j.heliyon.2023.e22688

**Published:** 2023-11-22

**Authors:** Emily J. Yang, Julian Fulton, Swabinash Swarnaraja, Cecile Carson

**Affiliations:** a California State University Sacramento, 6000 J St. Sacramento, CA 95819-6001, USA; b Folsom High School, 1655 Iron Point Rd, Folsom, CA 95630, USA; c Keep California Beautiful, 8665 S. Union Ave, Bakersfield, CA 93307, USA

**Keywords:** Machine learning, Citizen science, Community monitoring, Litter, Trash, Storm water

## Abstract

Machine learning (ML) and citizen science (CS) are increasingly prevalent and rapidly evolving approaches to studying and managing environmental challenges. Municipal and other governance actors can benefit from technology advances in ML and public engagement benefits of CS but must also address validity and other quality assurance concerns in their application to particular management contexts. In this article, we take up the pervasive challenge of urban litter to demonstrate how ML can support 10.13039/100013015CS by providing quality assurance in the regulatory context of California's stormwater program. We gave quantitative CS-collected data to five ML models to compare their predictions of a qualitative, site-specific, multiclass “Litter Index” score, an important regulatory metric typically only assessed by trained experts. XGBoost had the best outcome, with scores of 0.98 for accuracy, precision, recall and F-1. These strong results show that ML can provide a reliable complement to CS assessments and increase quality assurance in a regulatory context. To date, ML and CS have each contributed to litter management in novel ways and we find that their integration can provide important synergies with additional applications in other environmental management domains.

## Highlights

• Machine learning can help validate citizen science monitoring data in a regulatory context.
• XGBoost predicted qualitative litter assessments with scores of 0.98 for accuracy, precision, recall and F-1.
• Environmental management can be made more efficient by synergies between citizen science and machine learning.

## 1 Introduction

Citizen Science is an inclusive approach to scientific research and monitoring that brings together expert scientists in government and academia with non-expert participants to produce knowledge. CS activities range from crowdsourced data contributions through mobile phone apps to more-involved collaborations and co-creations with non-experts participating in research design, analysis, and dissemination [[1], [2], [3], [4], [5]]. As CS projects have proliferated in recent decades, more critical attention has been paid to their formal role in cutting-edge science and the management, regulatory and policy work of government agencies [6,7]. As government and academic scientists have come to rely more heavily on CS contributions to their projects, they have had to navigate debates around CS validity and reliability [8,9]. Generally, proponents of CS argue that it expands the potential for data collection and processing, leading to myriad public benefits, including better outcomes of scientists' goals a more informed and engaged public [10,11]. On the other hand, skeptics argue that non-expert participants’ lack of formal training and potential bias renders their contributions untrustworthy and may even erode the standing of science in society. Much work has gone into developing methods that address questions of validity, for example, through participant engagement, training and quality assurance procedures for how data are generated, managed and used for project goals [2,[12], [13], [14]].

A complementary approach is Machine Learning (ML), defined as “the science (and art) of programming computers so they can learn from data” [15]. Machine Learning is a subset of Artificial Intelligence and is a rapidly evolving field of computer science with various applications, including to support 10.13039/100013015CS approaches in environmental research and management. In this article, we ask “*how can machine learning* support *the role of* 10.13039/100013015*CS* *in environmental management*?” While this article focuses on litter management, we intend to inform environmental managers more broadly about the potential for ML to support 10.13039/100013015CS approaches, particularly in regulatory and other governance contexts. The remainder of this introduction describes existing literature on the intersections of a) ML and CS, b) ML and litter management and c) CS and litter management. Second, we describe a technical case study of ML applied to a CS-based litter monitoring project in the regulatory context of California's stormwater permitting program. We then discuss the contributions of our successful ML results to existing literature and the practical integration of ML and CS into the management of litter and other environmental domains.

### 1.1 Machine learning and citizen science

The intersection of ML and CS has emerged as a promising avenue for addressing various scientific challenges in a more efficient and cost-effective way [16]. Recent studies show that ML can significantly improve the quality of CS data, mostly imagery, by automating data processing, reducing human errors and increasing data accuracy. Langenkämper et al. [17] explored a combination of CS and deep learning-based classification of marine imagery to identify conditions under which large imagery datasets can be efficiently and reliably used for research. Jakuschona et al. [18] similarly evaluated various image-based species recognition models for the purpose of processing crowdsourced images in support of European invasive species policy.

ML studies have also attempted to address common CS challenges, for example, reducing barriers for non-experts. Wood et al. [19] demonstrate how ML-based bird recognition algorithms can reduce the time and effort required to identify bird species, making it more accessible for people with limited resources or expertise. Lotfian et al. [20] discuss such benefits in several research contexts and further explain that ML can enhance CS engagement by providing a better user experience and increased participant motivation. McClure et al. [21] also identify the engagement and technological synergies of CS-ML integration in ecological monitoring, and elaborate other considerations to help program managers optimize returns on allocation of limited resources.

While the integration of ML and CS offers numerous opportunities, some potential risks should also be considered. Franzen et al. [22] discuss the ethical implications of using ML in CS, emphasizing the importance of transparency, fairness and accountability. Additionally, Ponti & Seredko [23] explore the challenges of allocating tasks between humans and machines in CS projects, highlighting the need for effective communication and collaboration between all stakeholders. Overall, this literature shows a huge range of possibilities, albeit mostly within the application of imagery analysis, as well as challenges to work on in intersection of ML and CS. Our work contributes a novel application of ML using non-imagery data collected by citizen scientists during litter cleanups to assess how litter conditions change over time.

### 1.2 Machine learning and litter management

As with other ML studies, the main application of ML to litter management has been object detection in imagery, where litter is one type of object that is identified among other image objects and backgrounds. Such approaches can fill important data gaps, reduce costs and allocate litter management resources more efficiently. The use of Convolutional Neural Network (CNN) models for deep learning on trash images was proposed by Mittal et al. [24], who developed an Android app called SpotGarbage, which used CNN to produce a coarse-grained segmentation of garbage area and non-garbage area in imagery from heavily polluted regions. Other works have used CNN in various waste contexts, including to support identification and sorting in managed waste streams [25,26] as well as for in-situ litter monitoring using mounted cameras in vehicles [27] and surveillance videos [28]. To increase the imagery coverage for detecting litter in the environment using ML, unoccupied aerial vehicles (UAVs, aka drones) have also been used [[29], [30], [31]].

The ML and deep learning-based approach for object recognition relies on the images taken in various ways, such as pictures and videos. While this may be a cost-effective approach to expand the geographic scope and frequency of litter monitoring, it has its limitations. As discussed by Liu et al. [28] about this type of approach, “small-sized objects in picture, luminance changes, moving occlusions and public facilities in ground all cause unexpected problems for litter detection.” Compared to image recognition, CS-based walking surveys can provide more accurate quantitative assessment, especially in the following conditions in cities: 1) when trash is partially degraded and blended within dry grass or bark, 2) areas not visible from common camera placement, e.g. behind bushes, 3) items that are smaller than surrounding items, especially in a low light situation. Human surveyors can overcome these challenges and provide additional information to identify trash materials (e.g. plastic or metal) and other site features that can be critical for managers to develop solutions and allocate resources.

### 1.3 Citizen science and litter management

Organized litter cleanups along shorelines, creeks, roadways, and neighborhood parks are a common volunteer or service activity. Many such programs have been extended to include a 10.13039/100013015CS approach to support litter research. Kawabe et al. [32] reviewed 85 publications on CS in marine litter research, reporting that data collection comprised the bulk of CS participation but that methods and training were often not explicitly described. In contrast, Zorzo et al. [33] described an approach for integrating official monitoring and CS for beach litter in Spain, finding no significant difference between the data collected by the experience scientists and trained volunteers. Similarly, Bouzekry et al. [34] used CS data from trained schoolchildren in Morocco to assess beach conditions using established indices. Indeed, the benefits of CS contributions to litter research have been demonstrated in many contexts, including coastal assessments in India [35], China [36] and Norway [37], floating marine litter around Taiwan [38], and COVID-19 face mask litter along Canadian roadways [39]. Still, as Kawabe et al. [32] found, most CS contributions are limited to more objective, quantitative data such as tallies, weight or volume of collected trash items. None of these CS-litter studies leveraged ML capabilities to support 10.13039/100013015CS contribution of more advanced data collection or analysis. For example, qualitative assessments can be equally important to management objectives but may raise additional concerns about technical expertise and quality control when CS participants are involved.

The following technical case study describes a California CS-based litter management program that involves quantitative and qualitative data collection. Because both data types may be used in a regulatory context, various quality control measures are in place. Machine learning plays a central role and provides a novel innovation at the intersection of citizen science and litter management with applications for other environmental management domains.

## 2 Technical case study

### 2.1 Project Overview

The Trash Rapid Assessment Data Exchange (TRADE) is a publicly funded, publicly available tool to support the use of 10.13039/100013015CS data in order to monitor and manage the trash in stormwater systems. Water-born trash can found throughout aquatic ecosystems but is a particular concern in urban systems, among other “grand challenges” [3,40,41]. In California, municipal separate storm sewer systems (MS4s) are typically cities regulated by California's various permits to discharge stormwater into regulated waterways under section 402 (p) of the federal Clean Water Act. A statewide “trash amendments” policy modified these permits to require the elimination of trash from stormwater discharges by 2030 (in some cases sooner).

MS4s (permittees) must comply by either installing trash full-capture systems such as catch basins (“Track 1”) or demonstrating equivalent outcomes through non-structural controls such as street sweeping, litter campaigns and other institutional measures to prevent litter from entering the stormwater system in the first place (“Track 2”). Specific requirements vary by regional permits, but generally, Track 2 MS4s must conduct on-land assessments of trash conditions with sufficient frequency and coverage within their service areas to demonstrate progress. Thus, while Track 1 entails higher capital costs for MS4s, Track 2 can require a burdensome monitoring program that consumes staff time or contractual budgets. These challenges have led to compliance backsliding among under-resourced MS4s and a collective risk of not meeting the water quality objectives of the trash amendments.

Adjacent to this regulatory approach to litter management, volunteer- and service-based litter cleanups have been active around California and can help alleviate the MS4 monitoring burden under certain conditions. Typically, participants work in teams of two or three and are provided with a bucket, gloves and a safety briefing before being directed to a designated area for data collection and litter collection. Some cleanup events also include CS-style data collection, like counting collected trash items (e.g. plastic bags and straws) and logging data on paper or app-based surveys. While these quantitative CS datasets have been used to inform waste policy (e.g. plastic bag or straw bans) and track their effectiveness, they are not well-suited for regulatory use under California's stormwater permits. In particular, Track 2 compliance hinges on qualitative, rapid assessment of site trash conditions using a scale of Very High, High, Moderate, or Low, which requires more extensive training than is typically offered at volunteer cleanup events. Instead, city staff or contractors more often comprise the trained experts who can cover more sites across their jurisdiction, albeit without cleaning up trash, and later translate qualitative scores into quantitative metrics using research-based ranges of trash loading rates (gallons of trash per acre per year) [42].

TRADE takes a two-pronged approach to better suit CS-based activities to the regulatory needs of MS4s. First, it provides training for CS volunteers to develop expertise in qualitative, rapid trash assessment, which term the *Litter Index,*^1^ through a university-extension model that we describe in the discussion section. Second, TRADE includes several quality control measures to validate CS-based assessments. One important measure, which the rest of this case study focuses on, is the use of ML on the quantitative portion of CS data to predict qualitative Litter Index scores, which can then be compared to Litter Index scores entered in the field by CS volunteers.

TRADE data components consist of two data ingestion pathways, data processing, and data presentation. In the first ingestion pathway, we used *ESRI Survey 123 Connect* to build a cell phone application (“app”) that collects data from the field according to the schema used by our non-profit project partner, Keep California Beautiful (personal communication). This schema includes site attributes, a Litter Index score and itemized trash counts by groups, such as plastic, metal and paper, each of which has multiple item types, e.g. plastic bottles and plastic straws (see Appendix A). Volunteers can input the quantity of each trash item and upload pictures. The second pathway is for incorporating data collected outside of the TRADE project. We designed a data ingestion portal that allows authorized users to upload their data to our system. Data can be uploaded from other ArcGIS systems or from Excel files. All data transformation, computation, analysis and error handling in our system is conducted using the Python programming language. Through the task scheduling functionality of ArcGIS Online Notebook, data processing is scheduled to be executed automatically.

Role-Based Access Control is a security control approach that assigns users to roles and assigns roles with data access privileges. In our system, role-based access control is conducted through ArcGIS Hub [43]. Trained volunteers are authorized to submit field surveys. The file uploader role allows the use of the data ingestion portal to upload data. Permittees are divided into multiple groups. Each permittee can only edit its own survey data. In this way, data are securely controlled with different authorizations to avoid unauthorized modifications.

**Fig. 1:**
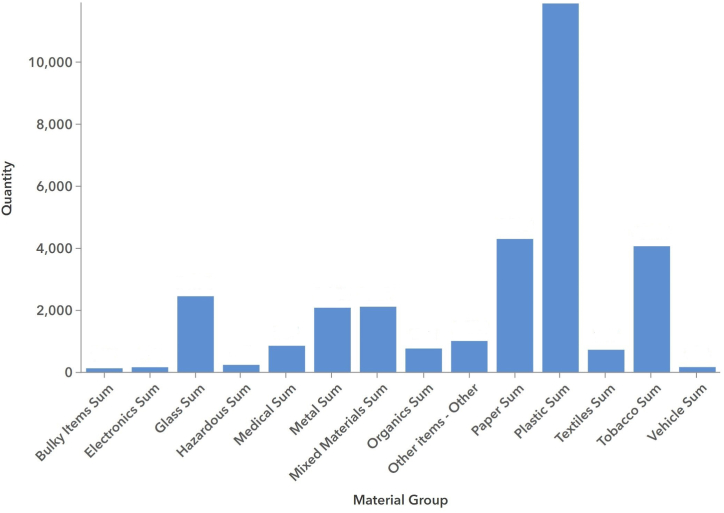
Example materials bar chart.

**Fig. 2:**
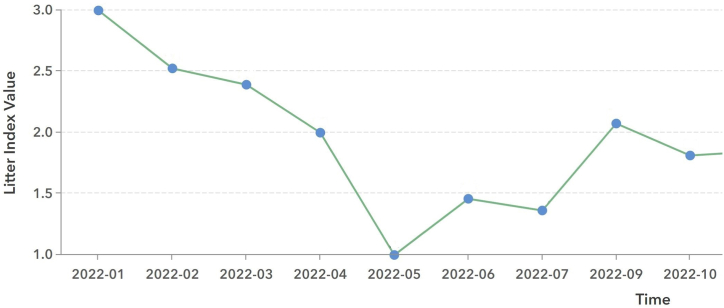
Litter Index change over time.

TRADE's data presentation system allows users to analyze the data through rich visualization. Users can filter the data to a date range and region of interest, for example, an individual survey location, a user-drawn polygon, or the entire MS4 jurisdiction. Fig. 1 shows an example bar chart for material groups. For example, the total number of bulky items is 147. Fig. 2 shows an example line chart of Litter Index that shows the changes over time, which is critical for demonstrating permit compliance.

**Fig. 3:**
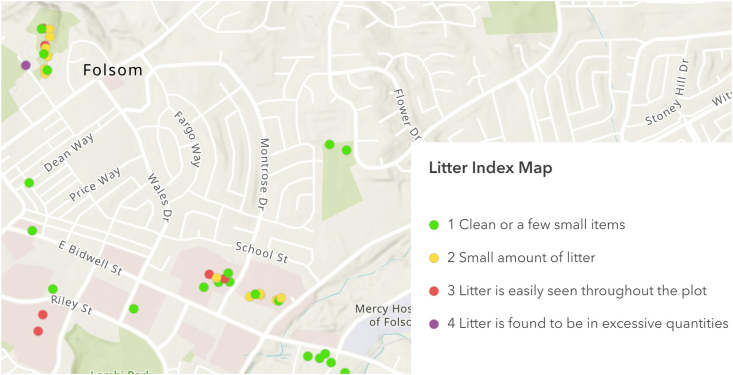
Example map showing individual surveys with litter index.

As shown in Fig. 3, surveys are also presented spatially on a map as points with different colors representing the Litter Index score: green for “1 Clean or a few small items”; yellow for “2 Small amount of litter”; red for “3 Litter is easily seen throughout the plot” and purple for “4 Litter is found to be in excessive quantities”.

### 2.2 Overview and Workflow

**Fig. 4:**
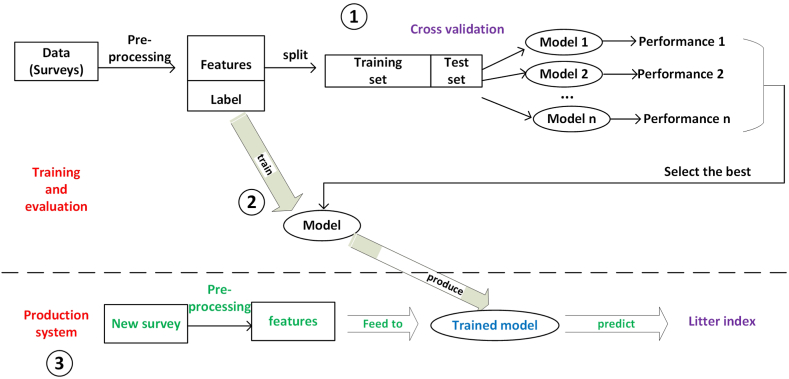
Overview of the execution flow.

To validate CS-based qualitative Litter Indexes, we use ML to predict Litter Indexes based on non-qualitative data. This section describes the execution flow of this process briefly, as shown in Fig. 4, and details are provided in subsequent sections.

The training and testing data originate from survey results collected by students and community volunteers in California from August 2021 to December 2022. Volunteers were trained on the Survey 123 app to tally collected trash items by and perform the qualitative Litter Index assessment. We cleaned the survey data by pre-processing and then separating the data into two parts: features and target. The target (called “label” in the training set) is the Litter Index value. The rest of the survey attributes that are useful for the prediction, such as number of plastic items, are the features. Domain experts in our team labeled the values of Litter Index for all surveys based on previous expertise on litter assessment, which serves as the “true” values, also called “labels”. After that, the data set was split into a training set and a testing set, and we selected a specific ML model, for example decision tree, then trained this model using the training set. Next, we used the test set to test the performance of this model. By using cross-validation (detailed in Section 3.3), we evaluated different models and selected the best performing model and used it in the production system. The final model chosen is XGBoost [44] (detailed in Section 3.4). Next, we used the whole data set, including features and labels, to train XGBoost.

In the production system that is hosted in ArcGIS online, when a new survey comes in, we pre-process the new survey to produce the features that have the same schema as the schema in training. Next, we feed the features into the trained XGBoost model. The trained model then predicts the Litter Index and the predicted value can be displayed on the dashboard automatically.

### 2.3 Data pre-processing, feature engineering and dimension reduction

The dataset contains 239 surveys conducted by volunteers under the TRADE project (see Supplementary Material for survey schema). Some questions in the surveys were not relevant to Litter Index prediction, such as number of volunteers, so they were removed. There are 62 types of trash items, such as plastic bottles, which were initially considered as features. There are also survey questions about if there are homeless camps and if the site is an illegal dump site, with three values of “yes active,” “yes inactive” and “no.” This type of categorical values, such as “no,” cannot be processed by the ML models directly, so we use one-hot encoding to replace the homeless camp attribute with three attributes: homeless_camp_yes_active, homeless_camp_yes_inactive, homeless_camp_no. For example, for the original “yes active” value in the survey, the encoding is [1,0,0]for [homeless_camp_yes_active, homeless_camp_yes_inactive, homeless_camp_no]. Our initial experiment was based on the features that include each type of trash item (e.g. plastic bottle, plastic straws, paper bags, electronic cigarettes, vehicle debris, aseptic container) and the one-hot encoded features of homeless camp (e.g. homeless_camp_yes_active) and illegal dump site. The performance measures, such as accuracy, are less than 0.85. The performance measures of this initial experience are shown in Appendix B.

One way to improve the performance is dimension reduction. For a small data set with less than 300 records and more than 60 dimensions (number of features), reducing dimensions can be a good solution in our case. The one-hot encodings on homeless camp and illegal dumping produce 6 dimensions but they actually do not associate with the Litter Index closely. For example, when there is an inactive homeless-camp, the score can be varied from 1 to 4, so homeless camp does not have a direct association with the Litter Index. As a result, we decided to remove homeless camp and illegal dumping from our features. Another observation is that the data set is sparse, which means there are many zeros in many trash items. We aggregate the counts of trash items (e.g. plastic bottle, plastic straws) into material group (e.g. plastic). There is a total of 14 material groups, so we reduce the dimension from 62 (trash items) to 14 (material groups).

When numerical attributes have very different scales, ML models may not perform well. It is typical to rescale all the values to the range from 0 to 1. For example, we can use the min-max scaling approach as follows to scale a value *v* to *u*, in which lowest is the lowest value in the data set and highest is the highest value in the data set, as shown in (1).

(1) *u= (v – lowest) / (highest - lowest)*

Outliers, abnormal high or low values in the data set, can make a difference in this approach. For example, if the highest value is abnormally high, the “u” value will be abnormally small. Instead of removing outliers to avoid this type of problem, we take a more practical approach. The lowest value can be 0 meaning no trash, which is straight-forward. In our case, Litter Index 4 means “Litter is found to be in excessive quantities”. So, whether there are 1000 plastic items or 10000 plastic items, it should be scored as 4. As a result, we set a ceiling variable whose value was given by the expert, as 200. That is, when an item exceeds 200, no matter how much more, the evaluation result will stay the same, which is Litter Index 4. In this way, when an item has an abnormally high value, this big number will not serve as the denominator to affect the scaling result. The same ceiling value was given to all material groups. So, instead of using the maximum value in (1), we use the ceiling value for min-max scaling, as shown in (2):

(2) *u= (v – lowest) / (ceiling - lowest)*

If the value of a feature is greater than the ceiling, then we assign the ceiling value to this feature before the scaling. As result, all values in the data set can be scaled properly from 0 to 1.

### 2.4 K-fold cross validation and performance measures

We split our data set into a training set and a test set according to a ratio, for example, 20 % of the data is in the test set. The training set is used to train a ML model and the test set is used to test the performance of the model. Instead of randomly putting a record either in the training set or the testing set, we use a stratified method to split the data according to the label that preserves the percentage of samples for each class. For example, 96 records have Litter Index 1, so 80 % of 96 records is in the training set and 20 % of the 96 records in the test set. Our data set is imbalanced, in which 40 % of the records are in the class of Litter Index 1. In an imbalanced multiple class classification problem like ours, stratified splitting produces more fair evaluations on models than random splitting.

If we split the data set multiple times, we will most likely get different training sets and test sets. The performance evaluation on one splitting result can be much better than the others. A better way to evaluate the performance is using K-fold cross validation (following [15]), where we divide the data into k number of folds and performance training and testing in k rounds. In the *i*th round, we take the *i*th fold as the test set and the rest of folds as the training set. By the end of the kth round, each fold has been used as a test set in a specific round. The final performance evaluation is based on the average performance of all rounds. We set the K value as 5, because our data set is small and we want to have enough data in the test set to evaluate the performance. When K is 5, in each run, 80 % data is for training and 20 % is for testing.

The commonly used performance measures for classification problems are accuracy, precision, recall and F1 score. Accuracy measures how many of the prediction results is correct, as shown in (3).

(3) $$Accuracy=\frac{True\;Positive+True\;Negative}{Total\;predictions}$$

Precision specifies that of the positives predicted, the percentage that is truly positive, as shown in (4).

(4) $$Precision=\frac{True\;Positive}{True\;Positive+False\;Positive}$$

Recall defines that of all the positive cases, percentage that is predicted positive, as shown in (5).

(5) $$Recall=\frac{True\;Positive}{True\;Positive+False\;Negative}$$

F1 score is the harmonic mean of precision and recall, as shown in (6). Since the harmonic mean gives much more weight to low values, a model will only get a high F1 score if both recall and precision are high.

(6) $$F1=2\;*\frac{precision*recall}{precision+recall}$$

Most of our models are from Scikit-learn, which is a widely used python library for ML. For multi-class classification problem, there is a “macro” score and a “weighted” score. “Macro” does not consider label imbalances like ours and produces a metric for each label and finds their unweighted mean. The “weighted” score considers class imbalance by calculating metrics for each label and then finding their average weighted by support, which is the number of true instances for each label.

In (7) for weighted precision, Pi is the precision of Class i. Si is the support of Class i, that is the total number of true instances in Class i.

(7) $$Weighted\;Precision=\frac{\sum_{1}^{n}Pi*Si}{\sum_{1}^{n}Si}$$

In (8), Ri is the recall of Class i. In (9), Fi is the F1 score of Class i.

(8) $$Weighted\;Recall=\frac{\sum_{1}^{n}Ri*Si}{\sum_{1}^{n}Si}$$

(9) $$Weighted\;F1=\frac{\sum_{1}^{n}Fi*Si}{\sum_{1}^{n}Si}$$

### 2.5 ML Model Selection

We have trained and tested the following models: K-Nearest Neighbor (KNN) classifier, Support Vector Machine (SVM) Classifier, Decision Tree Classifier, Random Forest Classifier, XGBoost (XGB) Classifier. Deep learning models can perform well for larger datasets. Our dataset is too small to fully utilize the benefit of it.

The KNN model classifies a data point by considering data grouping based on distance. After calculating the distance between a query data point and the other points, KNN selects the K number of points which are near to this query data point. Our k value is 3 points. Each data point is assigned to a class based on plurality voting, meaning it receives more votes than any other, which may not be more than half. When calculating the distance between a query point and another point, there are several distance measures. In our implementation using KNN [45], we use 'minowski' with p = 2, which results in the standard Euclidean distance. This is the default hyperparameter of KNeighborsCalssifier and it can be tuned to different values such as ‘manhattan.’ When presenting the query point Q as a vector of (q1, q2, …, qn) and the other point V as a vector of (v1,v2, …vn), the Minowski distance between Q and V is defined in (10). When p = 2, it is Euclidean distance; when p = 1, it is the Manhattan distance.

(10) $$Minowski\;distance={\left(\sum_{i=1}^{n}{\left|q_{i}-v_{i}\right|}^{p}\right)}^{1/p}$$

Support Vector Machine (SVM) is a good choice for small to medium sized datasets. Classes can be separated by a straight line in case of linear SVM classification with a margin. In our case, the data is not suitable for linear separation, so we add polynomial features to separate data based on curved lines. Soft margins are typically used for classification. There are different types of kernels such as Linear Kernel, Polynomial Kernel and Gaussian RBF Kernel. More details on the model and explanations can be found in Ref. [15]. We use Support Vector Classifier of the Scikit-Learn [46] to implement our classification task, in which we set a polynomial kernel with a degree of 6. Other parameters are: “coef0” is 1, “C” is 4, “gamma” is 1.

Decision Tree (DT) classifier uses a binary tree structure to make a prediction. Given an input X with n features (e.g. number of plastic items), the objective is to predict the class (e.g. Litter Index 1, 2, 3, or 4) based on X. The decision starts from the root of the tree. At each node of the tree, the value of X determines the direction of the movement towards the next tree node. Eventually, when a leaf note is reached, the decision is made, which is the classification result. More details on the model and explanations can be found in Ref. [15]. We use DecisionTreeClassifier in Scikit-Learn [47], in which the hyperparameter of max_depth is set to be 3 to restrict the maximum number of edges from a leaf to the root. This is the optimized max_depth during our hyperparameter tuning to minimize underfitting and overfitting.

An ensemble method makes predictions based on the answers from a group of predictors. The predictors can be different models. Random Forest (RF) is an ensemble of decision trees. RF trains a group of decision tree classifiers and compares the predictions of these decision trees. The classification result comes from the most votes. More details on the model and explanations can be found in Ref. [15]. We used RandomForestClassifier in Scikit-Learn [48]. The hyperparameter of “n_estimators” is 200 to specify the number of trees in the ensemble.

Boosting is a type of ensemble method that combines multiple weak learners into a strong one. It trains predictors in a sequential manner. Each subsequent predictor tries to correct its previous predictor. Gradient Boosting adjusts a new predictor based on the residual errors made by its predecessor. The residual for each data point is the distance between the predicted value and its actual value. More details about Gradient Boosting can be found in Ref. [15]. XGBoost is an implementation of Gradient Boosting, which is available in Ref. [44]. After hyperparameter tuning, in our final XGBoost classification, the hyperparameter of “max_depth” is set to 3 to restrict the depth of participating trees. The “n_estimators” hyperparameter is set to 5. In all the models, hyperparameters are manually tuned. The default hyperparameters are used unless specified explicitly above. The performance tuning results can be found in Appendix B.

### 2.6 Results

We have evaluated each of the models in Section 2.5 using the measures of accuracy, weighted precision, weighted recall and weighted F1 score, as detailed in Table 1. Decision Tree and XGBoost have better performance than KNN, SVM and Random Forest. We chose XGBoost as our final model as it outperforms all other models.

**Table 1:** performance comparison of different models.

	Accuracy	F1-Score (weighted)	Precision (Weighted)	Recall (Weighted)
KNN	0.958	0.957	0.961	0.958
SVM	0.933	0.932	0.940	0.933
Decision Tree	0.975	0.975	0.977	0.975
Random Forest	0.953	0.952	0.957	0.953
**XGBoost**	**0.979**	**0.979**	**0.981**	**0.979**

**Fig. 5:**
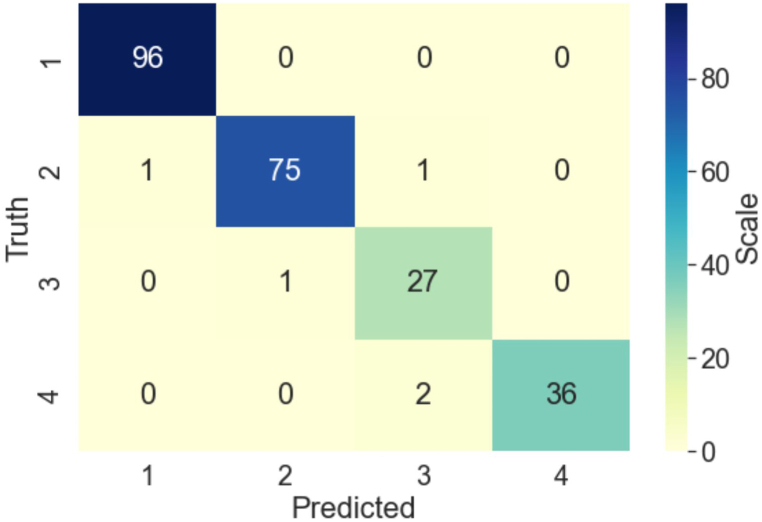
Confusion matrix of XGBoost.

Table 2 shows the details of prediction results within each class using XGBoost. We can see that the prediction of Litter Index 3 is the worst among all predictions. This class also has the smallest number of data records. To better see how the mistakes were made in the prediction, we can use a Confusion Matrix, as shown in Fig. 5. For example, there are 28 records in the class of Litter Index 3, but XGBoost predicted 30 records are in this class. Examining in the vertical direction, among the 30 predictions, 1 record actually belongs to Litter Index 2; 2 records actually belong to Litter Index 4. 27 records actually in Litter Index 3 were correctly predicted to be in the right class. Examining the horizontal direction, 1 record that actually belongs to Litter Index 3 was predicted by XGBoost as Litter Index 2.

**Table 2:** XGBoost performance in each class.

	Precision	Recall	F1-Score	Accuracy
Litter Index 1	0.99	1.00	0.99	0.98
Litter Index 2	0.99	0.97	0.98	
Litter Index 3	0.90	0.96	0.93	
Litter Index 4	1.00	0.95	0.97	
Macro average	0.97	0.97	0.97	
Weighted average	0.98	0.98	0.98

**Fig. 6:**
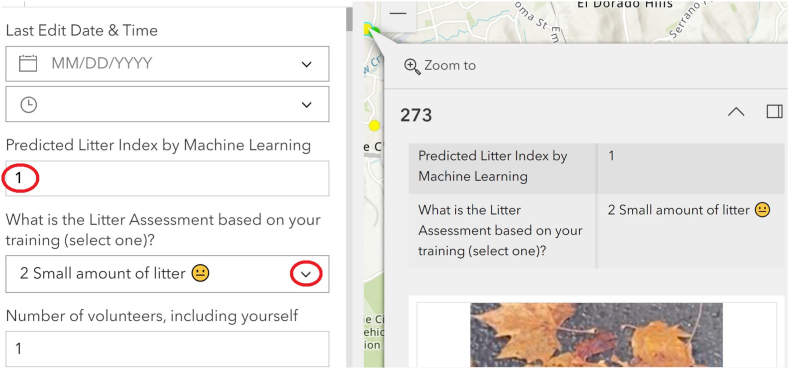
user interface to change a Litter Index value.

This work has been fully implemented in the ArcGIS online system. Once a new survey is submitted, the predicted Litter Index score will be presented with the rest of the survey data. An authorized user, such as city staff, can select the records in which the Litter Index scores provided by the volunteer and by the ML prediction are different by clicking a button. As shown in Fig. 6, an authorized user can check the details of the survey to decide the final Litter Index score. This provides a critical quality control step in the use of CS-based Litter Index scores for MS4 permit compliance purposes.

### 2.7 Data provenance

Another quality assurance measure to support 10.13039/100013015CS data use in a regulatory context for permit compliance is data provenance, or the ability to track the origin of data comes and its transformation steps through the system. Since this project accepts data from multiple sources, data provenance allows to track errors within the collected and processed data, identify the origin of data, understand how data was transformed, detect where the data has moved over time and aid in detecting data dependency [49]. Importantly, data provenance can be used to backtrack data and answer some critical questions such as who uploaded this data, where was this data originated from and when it was last modified [49].

In all ArcGIS feature layers, there is a field called GlobalID. GlobalID is a unique string that is generated as part of Esri's ArcGIS feature layer [50]. This field cannot be edited by neither the creator nor any organizational administrator. The GlobalID is unique throughout the entire Esri system and is not limited to a user account or particular organization [50]. The string includes a mix of digits between zero and nine and English alphabetical characters in lowercase. The combined mix of digits and English alphabetical characters create the unique string for each record in the ArcGIS feature layer [50].

Since the GlobalIDs are unique in ArcGIS system, this can be used as part of data provenance. In our feature layer, named as ETAP feature layer, we created a field named as reference ID. This field is used to keep the original identification of the data. When retrieving data from feature layers, the GlobalIDs for the data can be retrieved and stored. However, if the data is uploaded from an Excel spreadsheet, then GlobalIDs are absent. Therefore, a set of combined fields are utilized to generate a unique ID for each record in our feature layer. The combined fields include first and last name, username, date and time of uploading, email and ObjectID. Unlike the GlobalID, the ObjectID is unique for a dataset, but it is not unique across the ArcGIS system. Thus, using this field combination will generate a unique ID for each record in the ETAP feature layer that can be utilized to trace the origin of the uploaded data.

After a user uploads their data through the data ingestion portal, the data is processed and transformed. When retrieving data from the ingestion portal, the information is loaded into ArcGIS Notebooks for data transformation. However, the data uploaded by the user is never affected or altered. The ArcGIS Notebooks use a copy of the data for the data transformation. At the end, there will two sets of data where one of them was the original data uploaded by the user and the other data being the transformed data.

Tracking changes to the data will certainly add credibility and to track all errors within the collected and processed data. Our data is available to the public for viewing purposes; however, the data cannot be manipulated or altered by the public. Recall that our data is also shared with various permittee representatives who have the authority to alter the data. An exclusive web dashboard is created for each permittee where the data from their own area can be altered. For example, a representative from City of Folsom can modify Folsom data.

The changes and modifications made by the representative is completely and automatically tracked through our python scripts using ArcGIS Notebooks and ArcGIS feature layers. To track all changes made by various permittee representatives to the data that is exclusively available to their own permittee, a transaction layer is implemented using ArcGIS feature layer. The transaction layer includes all fields from ETAP feature layer and one additional field called “Last Edit Date & Time”. This field includes date and time information indicating when the information was modified for the record.

An ArcGIS Notebook was developed using Python scripts and scheduled to run every hour. The script was programmed to retrieve all data modified by various permittee representatives within the last hour. All modifications are stored without overwriting existing information. Therefore, a tuple that was edited multiple times will have multiple records in the transaction layer where each transaction reflects the changes made in that particular transaction. As a result, we can use the transaction layer to trace how a record is modified over time. In summary, data provenance provides a quality assurance measure to trace original data, understand data transformation, detect data dependency and bring credibility to the CS data as it is collected and presented in the dashboard.

## 3 Discussion

Our work shows that ML on quantitative CS data with XGBoost can be a strong predicter of qualitative Litter Indexes, with accuracy, precision, recall and F-1 scores of 0.98 (weighted average). These scores are higher than in other ML studies on imagery analysis, as discussed in Section 1.2 (except the work in Ref. [28] which also reported 0.98 accuracy), although there are several essential distinctions in these different ML applications. Other studies’ targeted problem with ML is litter detection; our goal uses litter data collected by humans to predict Litter Index. More importantly, the training datasets (i.e., imagery vs. survey data) are quite different in nature and not directly comparable in terms of performance.

**Fig. 7:**
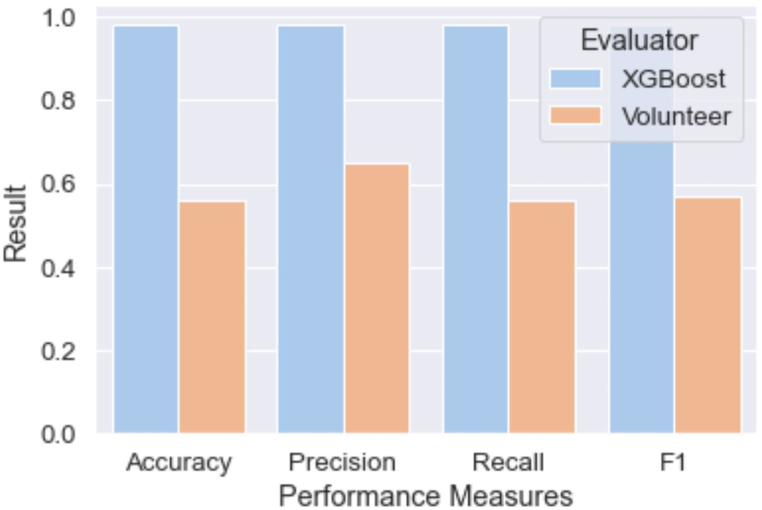
performance results of XGBoost vs. volunteer assessment.

A more useful comparison in the context of validating CS is to compare our ML results with the performance of actual volunteers' Litter Index assessments. For this, we evaluated the volunteers’ results using the same performance measures. We used the Litter Index scores provided by volunteers with the labeled values (truth) to calculate the following scores: Accuracy 0.560; Precision 0.646; Recall 0.560; and F1-score 0.572. Fig. 7 compares these results with XGBoost, which outperforms volunteer assessments by at least 33 % in all measures.

While ML prediction may outperform human assessment of Litter Indexes, it needs not supplant the role of CS in the regulatory context of litter management. Instead, our work shows that MS4s need not rely solely on a CS-volunteers’ Litter Index; instead, the ML-predicted Litter Index is a good reference for quality control reviewers to decide the final score. TRADE provides them that role as well as a data flag where ML and CS scores differ. Because of the high accuracy of our prediction, our approach can serve applications beyond CS, for example to quality control assessments conducted by city staff and consultants. Such experts may assign different Litter Assessment scores for similar site conditions, reflecting their experience, various observer biases, or other common sources of variance in qualitative assessment. ML-predicted Litter Indexes viewed alongside assessments from human experts or trained volunteers can provide an important quality assurance measure to more accurately track progress.

With CS-based research and monitoring approaches, trust in data acquisition and processing are common challenges that hinder its more widespread use. Our research provides an exemplary approach to use computer science to minimize trust issue in CS. Even though volunteers may be well-trained by field experts before they conduct assessments, providing additional quality control measures can reduce mistrust and ultimately the usability of CS data. In our application, using quantitative survey components like material and item counts, as well as site attributes, to generate qualitative outputs (Litter Index) helps to validate our CS approach.

Another benefit of the CS approach is tapping into the myriad benefits that engaging with community members can bring. For example, as mentioned in the study by Haarr et al. [37]) involving CS can reduce people's tendency to litter. This engagement-related outcome of CS has been noted in general environmental behaviors [51] and shows great promise for addressing urban litter and other environmental management challenges. Furthermore, in the context of California's stormwater program, public engagement and participation are stated requirements of stormwater permits and recognized “best management practices” that also yield better outcomes.

## 4 Conclusion

ML and CS are both increasingly prevalent and rapidly evolving approaches to research and management that cities can use to more efficiently and reliably monitor trash conditions and other such “grand challenges.” In practice, CS has a huge potential to provide “people power” but resulting data may face skepticism on grounds of validity, especially in a regulatory context. ML can provide important validation and address the assumed need for intensive training of volunteers who contribute qualitative assessments or other forms of data collection or research activities.

This paper presented our empirical study of using machine learning to increase CS validity in the context of trash monitoring for compliance with California's municipal stormwater permits. Although this is a limited context, public participation in trash issues will remain active and we expect to both advance and assess ML-CS approaches in future research. Similar research can contribute more generally to the nexus of ML and CS. First, our experimental approach needs to be applied in a broader variety of urban contexts, i.e. different land-use types where assessments take place and with varying levels of litter. Second, connecting our research to imagery-based ML approaches could be fruitful, for example using litter detection on pictures taken by CS volunteers and combining those results with other imagery sources like street sweeper-mounted cameras. Such research can help identify tasks where CS and ML can have complimentary or synergistic roles and contribute to more effective environmental management.

Advances at the nexus of ML and CS have the promise to improve efficiency of projects addressing management challenges while yielding multiple benefits to the public and expert participants. As innovations in ML and CS increase, we expect that their nexus will be a fruitful area of further research for scholars, as well as for managers and policymakers seeking novel approaches to solving environmental challenges.

## Funding

This work was supported by the 10.13039/100000139U.S. Environmental Protection Agency grant agreement OS-84009601.

## Data availability statement

Data associated with this study has been published and is publicly available at Mendeley Data at https://doi.org/10.17632/9dbcd876wj.1.

## CRediT authorship contribution statement

**Emily J. Yang:** Writing - review & editing, Writing - original draft, Visualization, Methodology, Investigation, Formal analysis, Data curation, Conceptualization. **Julian Fulton:** Writing - review & editing, Writing - original draft, Supervision, Resources, Project administration, Methodology, Investigation, Funding acquisition, Formal analysis, Conceptualization. **Swabinash Swarnaraja:** Writing - original draft, Validation, Investigation. **Cecile Carson:** Supervision, Methodology, Data curation.

## Declaration of competing interest

The authors declare the following financial interests/personal relationships which may be considered as potential competing interests: Julian Fulton reports financial support was provided by 10.13039/100000139U.S. Environmental Protection Agency, United States.

## Footnotes

1 like California's stormwater permits, we do not specify a particular method for arriving at 4-point scores, but the dominant one used in the stormwater sector is the on-land visual trash assessment (OVTA) method, available at https://eoainc.com/ovta/.
