# Supplementary material for: Machine learning to support citizen science in urban environmental management
Source: Heliyon. 2023 Nov 22;9(12):e22688. doi: 10.1016/j.heliyon.2023.e22688 (PMC10696195; doi:10.1016/j.heliyon.2023.e22688)

Appendix A

A1. Trash group and item type (from Keep California Beautiful, personal communication)

Trash Group	Trash Item Type
Bulk items	Appliance
	Camping Gear
	Furniture
	Mattress/Box Springs
	Shopping Carts
Electronics	Electronic cords
	Entertainment items
	Mobile Devices, Televisions, Computer
Glass	Beer, Wine, &Liquor
	Juice, Soda, Sports Drink, Water
	Broken glass or ceramic
Hazardous	Paint
	Batteries (non-vehicle)
	Chemicals and Pesticides
Medical	Medication bottles or tubes
	Needles/Sharps/Syringes
	Toiletries/ personal hygiene products
	Personal Protection Equipment
Metal	Beer, Wine, &Liquor
	Juice, Soda, Sports Drink, Water
	Food packaging, foil
	Straws, metal
	Metallic Waste
Mixed Materials	Aseptic container
	Gable top containers
	Condiment packages & Candy Wrappers
	Construction and demolition debris
Organics	Compostable Waste
	Food waste
	Human & Pet waste
Paper	Books & Magazines
	Cardboard & Signs
	Newspaper/ inserts
	Office paper/ mail

	Paper bags
	Paper cups
	Paper fast food service items
	Paper Straws
	Receipts
Plastic	Small Plastic Lids Rings Tabs
	Plastic Foam (Single Use Expanded)
	Plastic Foam (Coolers and Packaging)
	Plastic bags
	Plastic cups
	Plastic packaging film
	Single Use Food Service Items/Dishware
	Juice, Soda, Sports Drink, Water
	Retail/grocery bags
	Straws, plastic
	Beer, Wine & liquor
	Other plastic
Textiles	Clothing
	Rugs
	Sleeping Bags/Bedding
Tobacco	Cigarettes, Cigars & associated unused ends
	Cigarette or cigar containers and film
	Electronic cigarettes
Vehicle	Batteries
	Oil or vehicle fluids
	Tire tread
	Vehicle Debris
Other	Other items

A2. Performance

1. Performance measures before dimension reduction

	Accuracy	F1-Score (weighted)	Precision (Weighted)	Recall (Weighted)
KNN	0.585	0.538	0.588	0.585
SVM	0.826	0.823	0.839	0.826
Decision Tree	0.688	0.664	0.679	0.688
Random Forest	0.743	0.719	0.742	0.743
XGBoost	0.771	0.751	0.787	0.771

2. Performance tuning of KNN

	Accuracy	F1-Score (weighted)	Precision (Weighted)	Recall (Weighted)
n_neighbors=2 metric=default='minkowski'	0.932	0.930	0.936	0.932
n_neighbors=3 metric=default='minkowski'	**0.958**	**0.957**	**0.961**	**0.958**
n_neighbors=4 metric=default='minkowski'	0.945	0.944	0.948	0.945
n_neighbors=5 metric=default='minkowski'	0.945	0.944	0.948	0.945
n_neighbors=7 metric=default='minkowski'	0.949	0.948	0.951	0.949
n_neighbors=11 metric=default='minkowski'	0.937	0.935	0.938	0.937
n_neighbors=3 metric='manhattan'	0.936	0.935	0.940	0.936
n_neighbors=7 metric='manhattan'	0.937	0.935	0.939	0.937

3. Performance tuning of SVM

	Accuracy	F1-Score (weighted)	Precision (Weighted)	Recall (Weighted)
kernel="linear", degree=4, coef0=1, C=5, gamma=1	0.815	0.804	0.826	0.815
kernel="rbf", degree=3, coef0=1, C=5, gamma=1	0.891	0.887	0.901	0.891
kernel="poly", degree=4, coef0=1, C=5, gamma=1	0.929	0.928	0.937	0.929
kernel="poly", degree=6, coef0=1, C=4, gamma=1	**0.933**	**0.932**	**0.940**	**0.933**
kernel="poly", degree=10, coef0=1, C=5, gamma=1	0.929	0.928	0.941	0.929
kernel="poly", degree=8, coef0=1, C=5, gamma='scale'	0.912	0.909	0.918	0.918
kernel="poly", degree=8, coef0=1, C=5, gamma=1	0.925	0.923	0.936	0.925
kernel="poly", degree=7, coef0=1, C=4, gamma=1	0.932	0.931	0.940	0.933

4. Performance tuning of Decision Tree

	Accuracy	F1-Score (weighted)	Precision (Weighted)	Recall (Weighted)
max_depth=2, criterion=default='gini'	0.870	0.828	0.804	0.870
max_depth=3, criterion=default='gini' (More than one set of parameters has the same performance)	**0.975**	**0.975**	**0.977**	**0.975**
max_depth=4, criterion=default='gini'	0.971	0.971	0.972	0.971
max_depth=5, criterion=default='gini'	0.962	0.962	0.964	0.962
max_depth=6, criterion=default='gini'	0.962	0.962	0.964	0.962
max_depth=10, criterion=default='gini'	0.962	0.962	0.964	0.962
max_depth=50, criterion=default='gini'	0.962	0.962	0.964	0.962
max_depth=default=none, criterion=default='gini'	0.962	0.962	0.964	0.962
max_depth=3, criterion= ' entropy'	0.975	0.975	0.977	0.975
max_depth=4, criterion= ' entropy'	0.975	0.975	0.977	0.975
max_depth=10, criterion= ' entropy'	0.958	0.957	0.960	0.958

5. Performance tuning of Random Forest

	Accuracy	F1-Score (weighted)	Precision (Weighted)	Recall (Weighted)
n_estimators=5, max_depth=3	0.795	0.782	0.810	0.795
n_estimators=5, max_depth=10	0.870	0.865	0.878	0.870
n_estimators=10, max_depth=10	0.928	0.925	0.933	0.928
n_estimators=20, max_depth=20	0.937	0.935	0.943	0.937
n_estimators=40, max_depth=default=none	0.937	0.935	0.943	0.937
n_estimators=100, max_depth=100	0.957	0.956	0.961	0.957
n_estimators=200, max_depth=100	0.953	0.926	0.957	0.953
n_estimators=200, max_depth=default=none	**0.953**	**0.952**	**0.957**	**0.953**
n_estimators=300, max_depth=default=none	0.949	0.948	0.953	0.949

6. Performance tuning of XGBoost

	Accuracy	F1-Score (weighted)	Precision (Weighted)	Recall (Weighted)
max_depth=2, n_estimators=5	0.979	0.979	0.980	0.979
max_depth=3, n_estimators =5 (more than one set of parameters has the same performance)	**0.979**	**0.979**	**0.980**	**0.979**
max_depth=4, n_estimators =5	0.975	0.975	0.977	0.975
max_depth=2, n_estimators =5	0.979	0.979	0.980	0.979
max_depth=3, n_estimators =9	0.971	0.970	0.972	0.971
max_depth=3, n_estimators =7	0.979	0.979	0.980	0.979
max_depth=2, n_estimators =6	0.979	0.979	0.980	0.979
max_depth=6, n_estimators s=6	0.975	0.975	0.976	0.975
max_depth=3, n_estimators =10	0.970	0.970	0.972	0.971
max_depth=15, n_estimators =12	0.975	0.975	0.976	0.975
max_depth=2, n_estimators =2	0.979	0.979	0.980	0.979
max_depth=100, n_estimators =5	0.975	0.975	0.977	0.975
max_depth=3, n_estimators =4	0.979	0.979	0.980	0.979
max_depth=2, n_estimators =100	0.970	0.970	0.972	0.971
max_depth=2, n_estimators =100	0.970	0.970	0.972	0.971
max_depth=100, n_estimators =200	0.970	0.970	0.972	0.971

A3. Confusion Matrix

1. Decision Tree

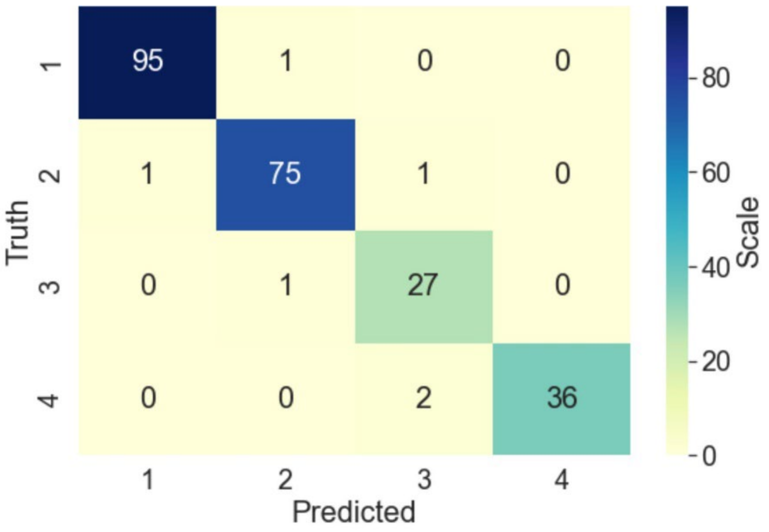

2. KNN

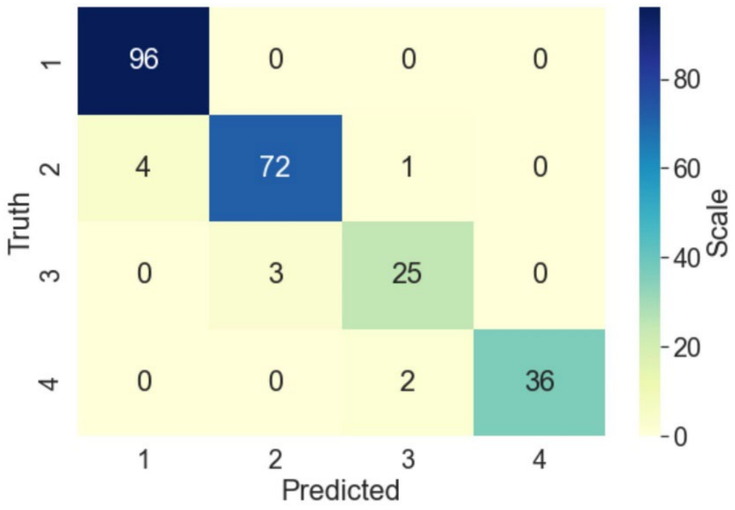

3. Random Forest

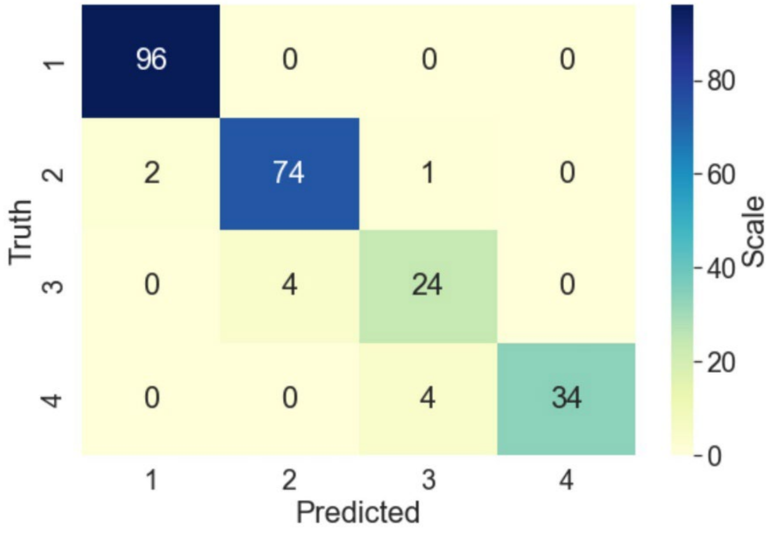

4. SVM

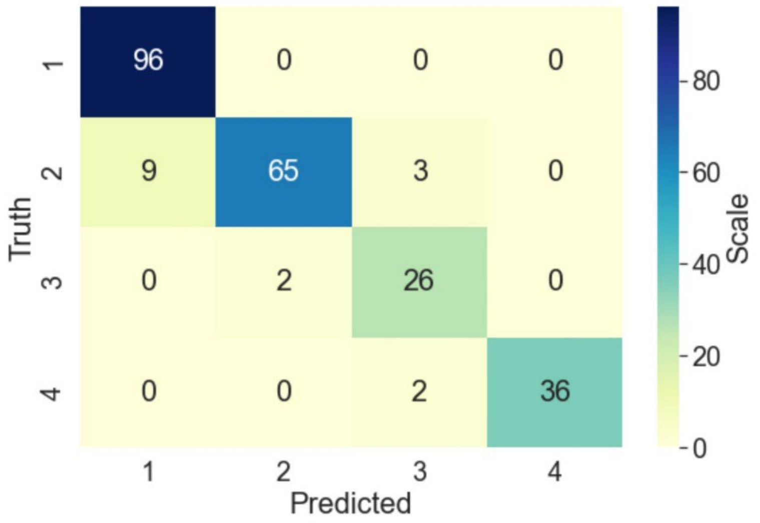

Supplement: Multimedia component 1 [file mmc1.pdf]
